# Cost Barriers to Health Services in U.S. Adults Before and After the Implementation of the Affordable Care Act

**DOI:** 10.7759/cureus.21905

**Published:** 2022-02-04

**Authors:** Danielle Kilchenstein, Jim E Banta, Jisoo Oh, Albin Grohar

**Affiliations:** 1 Public Health, Loma Linda University, Loma Linda, USA

**Keywords:** health care disparities, prescription medication, dental care, mental healthcare, healthcare services, affordable care act, cost barriers, insurance coverage, access to healthcare

## Abstract

Background: The Affordable Care Act (ACA) was passed in 2010 and implemented in 2014 in the United States (U.S.). It was partly intended to reduce the cost burden to health coverage and care.

Objective: To determine if ACA implementation reduced the odds of experiencing cost barriers to needed healthcare services for vulnerable groups.

Methodology: National Health Interview Survey Data from the Integrated Public Use Microdata Set (2011-2013; 2015-2017) were used to examine cost barriers to primary health, mental health, dental services, and prescription medications particularly for adults living in poverty, those of color, and unmarried individuals before and after implementation of the ACA. The study sample included 112,245 individuals, representing an annual average of 138 million adults (aged 26 to 64 years of age), including 59,367 survey respondents from 2011 to 2013 and 52,878 from 2015 to 2017.

Results: Pre/post-ACA, cost barriers to medical care decreased from 9.6% to 7.0% of adults, mental care from 3.0% to 2.4%, dental care 15.0 to 11.7%, and prescriptions from 9.9% to 7.0% (all comparisons p<.001). Survey design-adjusted regression results indicated significant decreases in the odds of experiencing cost barriers to physical, mental, dental health services and prescription medications after the implementation of the ACA for people living under 200% poverty, unmarried adults, and people of color. While the race was not a substantial barrier post-ACA, living in poverty and being unmarried remained the biggest predictors of cost barriers to services. Cost barriers for all services increased post ACA for adults with private coverage, and among older adults for prescription and dental services.

Conclusions: While the ACA was largely successful in reducing the number of uninsured adults in the U.S., remaining barriers suggest the need to strengthen the ACA and reduce cost barriers to healthcare services for everyone.

## Introduction

It is common for people in the United States to face bankruptcy due to high medical costs [1,2]. Cost barriers for receiving health care services are especially challenging for marginalized groups. While the Affordable Care Act (ACA) expanded coverage to people previously uninsured, it did not ensure newly insured populations understood coverage and out-of-pocket costs and were able to navigate the healthcare system or guarantee providers within close proximity [3].

The race is a contributing factor to experiences of cost barriers, though race disparities narrowed between whites and people of color on key health indicators, such as delaying care due to costs [4]. However, racial disparities were still wide after the implementation of the ACA, especially for those remaining uninsured [5]. Health coverage is a significant predictor of access but does not explain all racial disparities in access to care [6].

Social factors such as marital status are important influences on cost barriers to primary healthcare. For example, single mothers may face challenges to income stability as a result of child care and other costs that impede the ability to actively stay employed and maintain health coverage [7]. Further, studies show that unmarried people tend to delay medical treatments due to cost [8,9].

It is important to understand the individual and contextual factors impacting cost barriers to healthcare services in relation to the ACA, particularly related to marginalized groups such as people living below poverty levels, people of color, and unmarried adults in order to alleviate barriers and improve population health broadly. This research aimed to narrow the gaps in the literature by exploring cost barriers to care for marginalized groups by examining access across multiple domains of care before and after the ACA. Furthermore, to investigate associations between poverty status, race, and marital status and cost barriers to physical, mental, dental services, and prescription medications of adults aged 26 to 64 years old before (2011-2017) and after (2015-2017) implementation of the ACA in 2014.

The behavioral model of health services use

Andersen’s Behavioral Model of Health Services Use has been applied to multiple health outcomes and postulates that individual and contextual factors associated with health service use can be grouped into predisposing characteristics, enabling factors, and need categories [10-12]. This study applies the Andersen Model to examine inadequate health access through experiences of cost barriers to services and aims to determine how race, poverty, and marital factors are associated in relation to the ACA. Of particular interest is whether the ACA was associated with increased access to equitable care.

## Materials and methods

Data source

A cross-sectional analysis was completed using Integrated Public-Use Microdata Set (IPUMS) as the source of National Health Interview Survey (NHIS) data [13]. For this study, survey responses were combined into two time-based groups (2011-2013 and 2015-2017) to examine associations before and after ACA implementation.

Exclusion criteria were age 25 years and younger, people above age 65, non-citizens, and active-duty Armed Forces personnel. By definition, the NHIS excludes residents in long-term care facilities, persons in correctional facilities, and U.S. nationals living abroad.

Study measures

The cost barrier outcome measures from NHIS were: (1) needed but could not afford primary healthcare services during the past 12 months (yes/no); needed but could not afford mental (2), dental services (3), and prescription medications (4) during the past 12 months (yes/no). Main study variables included race and ethnicity (based on NHIS definitions of White, Latino, Black, Asian, American Indian/Alaskan Native, multiple/other races), household income (collapsed to less than 100% FPL, 100%-199% FPL, 200% FPL and over), and marital status (married, widowed, divorced, separated, never married).

Additional contextual variables included gender (male and female), age (individual years grouped into 26-35, 36-45, 46-54, 55-64 years of age), an education level (collapsed to no high school diploma, high school graduate, some college, college graduate, graduate school), geography (four Census regions of residence), employment status (had a job last week, none last week but had a job last 12 mo., none last week and none last 12 mo., never worked), public and private insurance coverage, and health status (excellent, very good, good, fair, poor). Psychological distress (no/low distress, moderate distress, severe distress) was used for the analysis of access to mental health services [14]. None of the other three outcomes had a survey question that so directly asked about the perceived need for that service.

Statistical analysis

After applying exclusion criteria there were 115,201 sample adults. Of these 2,956 (2.0%) had missing data for key independent variables, particularly poverty, education, marital status, and health status. As approximately 5% of adults had unknown income, this analysis included a category for unknown income rather than excluding those cases. The distribution of individual-level variables between people with missing values and those with complete values were similar. The final sample size included 112,245 individuals, with 59,367 survey respondents before the ACA and 52,878 after the ACA.

Descriptive analysis using cross-tabulation with design-adjusted Chi-square for categorical variables and both univariate and multivariable regression models were utilized to examine the associations between the independent and dependent study variables. Poverty status, educational attainment, employment status, and health status had Pearson r correlations around 0.3, indicating non-problematic collinearity. Statistical analysis was conducted using SAS 9.4 (SAS Institute, Cary, NC).

## Results

As seen in Table 1, there were 59,367 adult respondents from 2011 to 2013 and 52,878 from 2015 to 2017, representing 138 million on average annually. Chi-Square testing revealed statistically significant differences in race, ethnicity, poverty status, marital status, age, educational attainment, employment status, needed could not afford primary medical, dental, mental services, and public and private health coverage. Approximately 20% were persons of color, around 23% earned below 200% of the Federal Poverty Level (FPL), and roughly 40% were unmarried. Those who needed but could not afford medical care in the past 12 months dropped from 9.7% before the ACA to 7.0% after implementation (p<.001). Likewise, needing but could not afford mental care dropped from 3.1% to 2.4% after 2014, and not able to afford dental care from 15.1% to 11.7% (p<.001). See Table 1 for a full list of variables and survey respondent characteristics.

**Table 1:** Demographic characteristics of U.S. adults aged 26-64 before and after the implementation of the affordable care act (NHIS IPUMS) (n = 112,245) Notes: Data are from National Health Interview Survey from Integrated Public Use Microdata Set (IPUMS) 2011-2017 [13] *Variable with p-value <.001 from binary analysis using a Chi-square test Abbreviations: (Est. Pop. % [95% CI]): Estimated population percentage and 95% confidence interval

	Before ACA 2011-2013	After ACA 2015-2017
Estimated Annual N: Weighted Frequency	N = 313,300,000	N = 322,600,000
Survey n:	n = 59,367	n = 52,878
Characteristics	Est. Pop % (95% CI)	Est. Pop % (95% CI)
*Race		
White	80.4 (79.8, 81.0)	78.8 (77.9, 79.7)
Black/African American	12.7 (12.1, 13.2)	13.1 (12.4, 13.8)
Amer. Indian/Alaskan Native	0.7 (0.6, 0.9)	1.0 (0.7, 1.2)
Asian	4.3 (4.0, 4.5)	4.9 (4.5, 5.2)
Hispanic	10.2 (9.8, 10.6)	11.9 (11.1, 12.7)
Other Race	1.7 (1.5, 1.8)	2.0 (1.9, 2.2)
Ethnicity		
Hispanic	10.1 (9.7, 10.6)	11.8 (11.0, 12.6)
Non-Hispanic	89.8 (89.3, 90.2)	88.1 (87.3, 88.9)
*Poverty Status		
Less than 100% FPL	9.8 (9.4, 10.2)	9.0 (8.6, 9.3)
100-199% FPL	13.2 (12.8, 13.6)	14.0 (13.6, 14.5)
200% FPL and Over	70.7 (70.1, 71.4)	73.0 (72.3, 73.7)
Unknown FPL	6.1 (5.8, 6.4)	3.8 (3.5, 4.1)
*Marital Status		
Married	60.6 (60.00, 61.30)	60.3 (59.7, 61.0)
Widowed	2.1 (2.00, 2.27)	2.1 (2.0, 2.3)
Divorced	14.3 (13.99, 14.69)	13.7 (13.3, 14.0)
Separated	2.8 (2.72, 3.02)	2.5 (2.3, 2.7)
Never Married	19.9 (19.51, 20.45)	21.1 (20.6, 21.7)
*Age		
26-34	22.4 (21.9, 22.9)	23.1 (22.6, 23.6)
35-44	23.9 (23.5, 24.3)	23.6 (23.1, 24.1)
45-54	28.1 (27.6, 28.6)	26.4 (25.9, 26.9)
55-64	25.4 (25.0, 25.9)	26.7 (26.2, 27.2)
Gender		
Male	48.6 (48.1, 49.1)	48.5 (48.0, 49.1)
Female	51.3 (50.8, 51.8)	51.41 (50.8, 51.9)
*Educational Attainment		
No High School Diploma	9.1 (8.7, 9.4)	8.2 (7.8, 8.5)
High School Grad	24.9 (24.4, 25.5)	22.7 (22.2, 23.3)
Some College	32.2 (31.7, 32.7)	31.2 (30.5, 31.8)
College Grad	21.8 (21.3, 22.3)	23.7 (23.1, 24.3)
Graduate School	11.8 (11.3, 12.2)	14.0 (13.4, 14.5)
*Employment		
Had Job Last Week	72.8 (72.2, 73.4)	75.0 (74.4, 75.5)
No Job Last Week, Had Job P ast 12 Mo.	6.0 (5.7, 6.2)	5.2 (4.9, 5.4)
No Job Last Week, None P ast 12 Mo.	18.8 (18.3, 19.3)	17.3 (16.8, 17.7)
Never Worked	2.2 (2.0, 2.4)	2.4 (2.2, 2.6)
Health Status		
Excellent	28.4 (27.8, 28.9)	27.8 (27.2, 28.4)
Very Good	33.0 (32.5, 33.5)	33.7 (33.2, 34.3)
Good	25.7 (25.2, 26.1)	26.0 (25.4, 26.5)
Fair/Poor	12.8 (12.3, 13.2)	12.3 (11.9, 12.7)
Psychological Distress		
Serious Distress	3.9 (3.6, 4.1)	3.8 (3.6, 4.0)
Moderate Distress	8.9 (8.6, 9.2)	9.6 (9.2, 9.9)
No or Low Distress	85.8 (85.4, 86.3)	83.7 (83.2, 84.2)
Region of Residence		
Northeast	17.8 (17.2, 18.5)	18.0 (16.8, 19.2)
NorthCentral/Midwest	23.8 (23.1, 24.5)	23.1 (22.0, 24.0)
South	36.6 (35.8, 37.4)	36.4 (34.9, 37.9)
West	21.6 (20.8, 22.3)	22.4 (21.1, 23.7)
*Needed But Could Not Afford Medical Care Past 12 Mo.		
Yes	9.6 (9.3, 9.9)	7.0 (6.7, 7.3)
No	90.3 (90.0, 90.6)	92.9 (92.6, 93.2)
*Needed But Could Not Afford Mental Care Past 12 Mo.		
Yes	3.0 (2.8, 3.2)	2.4 (2.2, 2.6)
No	96.9 (96.7, 97.1)	97.5 (97.3, 97.7)
*Needed But Could Not Afford Dental Care Past 12 Mo.		
Yes	15.0 (14.6, 15.5)	11.7 (11.2, 12.1)
No	84.9 (84.4, 85.3)	88.2 (87.8, 88.7)
Needed But Could Not Afford Medications Past 12 Mo.		
Yes	9.9 (9.5, 10.2)	7.0 (6.7, 7.3)
No	90.0 (89.7, 90.4)	92.9 (92.6, 93.2)
*Medicaid/Public Insurance Coverage		
Yes	9.3 (9.0, 9.9)	12.6 (12.1, 13.1)
No	90.6 (90.2, 90.9)	87.3 (86.8, 87.8)
*Private Insurance Coverage		
Yes	70.1 (69.4, 70.7)	73.2 (72.6, 73.9)
No	29.8 (29.2, 30.5)	26.7 (26.0, 27.3)

The univariate logistic regression of Table 2 shows that racial groups (except Asians) had higher odds compared to whites; people living under 200% poverty experienced three to four times higher odds compared to people above 200% FPL, and unmarried people had higher odds compared to their married counterparts.

**Table 2:** Unadjusted multiple logistic regression analysis of the association of cost barriers to health services on poverty, race, ethnicity, and marital status before and after the implementation of the ACA (2011-2013; 2015-2017) Notes: Pre-ACA = 2011-2013; Post-ACA = 2015-2017; Implementation year 2014 was excluded Abbreviations: OR = Odds Ratio, CI = Confidence Interval, Ref = Reference Category p<.05*; p<.005**; p<.0001*** Source: Author’s analysis from the National Health Interview Survey from Integrated Public Use Microdata Set (IPUMS) 2010-2017 [13]

	Needed But Could Not Afford Medical Care	Needed But Could Not Afford Prescription Meds	Needed But Could Not Afford Mental Care	Needed But Could Not Afford Dental Care
	OR (95% CI)	OR (95% CI)	OR (95% CI)	OR (95% CI)
Race				
White (Ref)	1.00	1.00	1.00	1.00
Black/African American	1.43 (1.33, 1.54)***	1.47 (1.37, 1.58)***	0.84 (0.74, 0.95)*	1.20 (1.13,1.28)***
American Indian/Alaskan Native	1.40 (1.13, 1.74)**	1.30 (1.01, 1.67)*	0.78 (0.51, 1.19)	1.21 (0.99, 1.47)*
Asian	0.49 (0.42, 0.58)***	0.45 (0.37, 0.54)***	0.34 (0.25, 0.45)***	0.60 (0.52, 0.68)***
Other Race	1.88 (1.60, 2.21)***	1.68 (1.44, 1.96)***	2.02 (1.57, 2.61)***	1.71 (1.51, 1.95)***
Ethnicity				
Non-Hispanic (Ref)	1.00	1.00	1.00	1.00
Hispanic	1.14 (1.05, 1.24)**	1.26 (1.16, 1.37)***	0.97 (0.83, 1.12)	1.25 (1.16, 1.34)***
Poverty Status				
Above 200% FPL (Ref)	1.00	1.00	1.00	1.00
100-199% FPL	4.11 (3.85, 4.40)***	3.92 (3.67, 4.19)***	2.99 (2.68, 3.33)***	3.75 (3.55, 3.98)***
<100% FPL	4.82 (4.49, 5.16)***	4.92 (4.61, 5.24)***	4.46 (3.98, 5.01)***	4.66 (4.40, 4.93)***
Marital Status				
Married (Ref)	1.00	1.00	1.00	1.00
Widowed	2.59 (2.26, 2.97)***	2.46 (2.16, 2.81)***	2.65 (2.10, 3.34)***	2.41 (2.15, 2.69)***
Divorced	3.01 (2.81, 3.22)***	2.43 (2.26, 2.61)***	2.98 (2.64, 3.35)***	2.37 (2.24, 2.52)***
Separated	3.16 (2.84, 3.53)***	3.19 (2.83, 3.58)***	3.48 (2.89, 4.18)***	2.85 (2.58, 3.15)***
Never Married	2.23 (2.08, 2.38)***	1.68 (1.57, 1.80)***	2.23 (2.00, 2.49)***	1.83 (1.73, 1.94)***

Table 3 shows the multivariable regression results indicating that the ACA reduced the odds of adults experiencing cost barriers to medical care and prescription medications (post-ACA OR=0.81, 95% CI=0.76, 0.86, p<.0001) for medical care, and OR=0.76, 95% CI=0.71, 0.81, p<.0001 for medications compared to pre-ACA 2011-2013). Though no significant reduction of odds for cost barriers to medical care and prescriptions for people living under poverty and unmarried adults, there was a 9%-14% reduction in odds for people of color. Compared to people living above 200% FPL, adults with incomes below that threshold were two times more likely to experience cost barriers to needed medical care. Divorced, separated, and never married people also had higher odds of experiencing cost barriers to medical care compared to married adults.

**Table 3:** Multiple logistic regression analysis of the association between individual characteristics and cost barriers to primary medical and prescription medications before and after the implementation of the ACA Abbreviations: Ref = Reference Category, OR = Odds Ratio, CI = Confidence Interval Pre-ACA = 2011-2013; Post-ACA = 2015-2017; implementation year 2014 was excluded Source: Author’s analysis from the National Health Interview Survey from Integrated Public Use Microdata Set (IPUMS) 2010-2017 excluding 2014 [13]

	Needed But Could Not Afford Primary Medical Care		Needed But Could Not Afford Prescription Medications	
	OR (95% CI)	P-value	OR (95% CI)	P-value
Pre-ACA (Ref)	1.00		1.00	
Post-ACA	0.81 (0.76, 0.86)	< .0001	0.76 (0.71, 0.81)	< .0001
Race				
White (Ref)	1.00		1.00	
Black/African American	0.86 (0.79, 0.92)	.0001	0.91 (0.84, 0.98)	.02
American Indian/Alaskan Native	0.63 (0.49, 0.82)	.0006	0.64 (0.48, 0.84)	.002
Asian	0.57 (0.47, 0.68)	< .0001	0.55 (0.45, 0.68)	< .0001
Other Race	1.32 (1.11, 1.58)	.002	1.19 (1.01, 1.42)	.04
Ethnicity				
Non-Hispanic (Ref)	1.00		1.00	
Hispanic	0.85 (0.77, 0.94)	.001	0.99 (0.90, 1.09)	.86
Poverty Status				
>200% FPL (Ref)	1.00		1.00	
100-199% FPL	2.03 (1.87, 2.21)	< .0001	1.88 (1.73, 2.04)	< .0001
<100% FPL	2.12 (1.91, 2.35)	< .0001	1.88 (1.71, 2.06)	< .0001
Marital Status				
Married (Ref)	1.00		1.00	
Widowed	1.39 (1.18, 1.63)	< .0001	1.25 (1.07, 1.45)	.003
Divorced	1.79 (1.66, 1.93)	< .0001	1.43 (1.32, 1.55)	< .0001
Separated	1.51 (1.33, 1.72)	< .0001	1.42 (1.24, 1.62)	< .0001
Never Married	1.57 (1.45, 1.69)	< .0001	1.14 (1.06, 1.23)	.0005
Age				
26-34 (Ref)	1.00		1.00	
35-44	1.09 (1.00, 1.19)	.05	0.94 (0.86, 1.03)	.12
45-54	1.07 (0.98, 1.17)	.12	0.95 (0.87, 1.03)	.27
55-64	0.82 (0.75, 0.90)	< .0001	0.66 (0.60, 0.72)	< .0001
Gender				
Female (Ref)	1.00		1.00	
Male	0.75 (0.71, 0.80)	< .0001	0.62 (0.58, 0.66)	< .0001
Educational Attainment				
No HS Diploma (Ref)	1.00		1.00	
High School Grad	0.95 (0.86, 1.05)	.38	0.83 (0.76, 0.91)	.0002
Some College	1.14 (1.03, 1.26)	.008	1.00 (0.90, 1.10)	.99
College Grad	0.84 (0.75, 0.95)	.007	0.70 (0.62, 0.78)	< .0001
Graduate School	0.74 (0.63, 0.86)	.0002	0.50 (0.43, 0.59)	< .0001
Employment				
Had Job Last Week (Ref)	1.00		1.00	
No Job Last Week, Had Job Past 12 Mo.	1.26 (1.14, 1.40)	< .0001	1.52 (1.35, 1.71)	< .0001
No Job Last Week, None Past 12 Mo.	0.72 (0.66, 0.78)	< .0001	1.05 (0.96, 1.15)	.24
Never Worked	0.42 (0.34, 0.51)	< .0001	0.52 (0.43, 0.62)	< .0001
Health Status				
Excellent (Ref)	1.00		1.00	
Very Good	1.40 (1.28, 1.54)	< .0001	1.55 (1.39, 1.72)	< .0001
Good	2.57 (2.35, 2.82)	< .0001	2.88 (2.62, 3.17)	< .0001
Fair/Poor	4.92 (4.43, 5.46)	< .0001	6.10 (5.42, 6.87)	< .0001
Region of Residence				
South (Ref)	1.00		1.00	
NorthCentral/Midwest	0.96 (0.88, 1.04)	.35	0.94 (0.87, 1.02)	.16
Northeast	0.78 (0.71, 0.85)	< .0001	0.76 (0.69, 0.84)	< .0001
West	1.08 (1.00, 1.17)	.05	0.91 (0.84, 0.99)	.034
Public Coverage				
No (Ref)	1.00		1.00	
Yes	0.22 (0.20, 0.24)	< .0001	0.37 (0.33, 0.40)	< .0001
Private Coverage				
No (Ref)	1.00		1.00	
Yes	0.22 (0.21, 0.24)	< .0001	0.35 (0.33, 0.38)	< .0001

Self-reported cost barriers to prescription medication showed similar results to medical care. Odds ratios in the multivariable regression for adults of color were 9%-45% lower compared to whites; however, people identifying as other races had 19% higher odds of cost barriers to medications. Additionally, people living below 200% poverty had 88% higher odds of experiencing cost barriers to medications compared to those above 200% FPL. Divorced and separated adults had increased odds of cost barriers to prescriptions compared to married adults. Refer to Table 3 for results on additional variables.

The results of Table 4 show that post ACA (2011-2013) resulted in a 16%-20% reduction in odds of experiencing cost barriers to mental and dental care (post ACA OR=0.84, 95% CI=0.76, 0.93, p=.0012 for mental care; and post ACA OR=0.80, 95% CI=0.76, 0.85, p<.0001 for dental care). The odds of experiencing cost barriers to needed mental and dental services were also significantly higher (between 27% and97%) for people living under poverty and those who were unmarried (between 24% and 70%), while experiencing 26%-66% lower odds for race and ethnicity, except for adults in the other race category. 

**Table 4:** Multiple logistic regression analysis of the association between individual characteristics and cost barriers to mental care and dental care before and after the implementation of the ACA Abbreviations: OR = Odds Ratio, CI = Confidence Interval, Ref = Reference Category Pre-ACA = 2011-2013; Post-ACA = 2015-2017; implementation year 2014 Source: Author’s analysis from the National Health Interview Survey from Integrated Public Use Microdata Set (IPUMS) 2010-2017 [13]

	Needed But Could Not Afford Mental Care		Needed But Could Not Afford Dental Care	
	OR (95% CI)	P-value	OR (95% CI)	P-value
Pre-ACA (Ref)	1.00		1.00	
Post-ACA	0.84 (0.76, 0.93)	.0012	0.80 (0.76, 0.85)	< .0001
Race				
White (Ref)	1.00		1.00	
Black/African American	0.64 (0.56, 0.73)	< .0001	0.74 (0.69, 0.80)	< .0001
American Indian/Alaskan Native	0.34 (0.21, 0.56)	< .0001	0.55 (0.43, 0.69)	< .0001
Asian	0.41 (0.30, 0.55)	< .0001	0.65 (0.56, 0.75)	< .0001
Other Race	1.11 (0.84, 1.47)	.43	1.17 (1.01, 1.36)	.03
Ethnicity				
Non-Hispanic (Ref)	1.00		1.00	
Hispanic	0.78 (0.66, 0.92)	.0033	0.89 (0.81, 0.97)	.0091
Poverty Status				
>200% FPL (Ref)	1.00		1.00	
100-199% FPL	1.27 (1.11, 1.46)	.0005	1.97 (1.83, 2.11)	< .0001
<100% FPL	1.40 (1.18, 1.66)	< .0001	1.97 (1.82, 2.13)	< .0001
Marital Status				
Married (Ref)	1.00		1.00	
Widowed	1.38 (1.07, 1.79)	.01	1.32 (1.16, 1.50)	< .0001
Divorced	1.70 (1.49, 1.95)	< .0001	1.46 (1.37, 1.56)	< .0001
Separated	1.49 (1.19, 1.87)	.0005	1.37 (1.23, 1.53)	< .0001
Never Married	1.44 (1.27, 1.63)	< .0001	1.24 (1.16, 1.32)	< .0001
Age				
26-34 (Ref)	1.00		1.00	
35-44	0.86 (0.76, 0.99)	.04	0.91 (0.85, 0.97)	.005
45-54	0.72 (0.63, 0.83)	< .0001	0.87 (0.82, 0.95)	.001
55-64	0.43 (0.36, 0.50)	< .0001	0.73 (0.68, 0.79)	< .0001
Gender				
Female (Ref)	1.00		1.00	
Male	0.61 (0.55, 0.69)	< .0001	0.67 (0.64, 0.71)	< .0001
Educational Attainment				
No HS Diploma (Ref)	1.00		1.00	
High School Grad	0.93 (0.78, 1.10)	.41	0.98 (0.90, 1.07)	.79
Some College	1.23 (1.03, 1.46)	.02	1.21 (1.11, 1.33)	< .0001
College Grad	1.16 (0.95, 1.41)	.13	0.90 (0.81, 1.00)	.06
Graduate School	1.33 (1.06, 1.66)	.01	0.72 (0.62, 0.82)	< .0001
Employment				
Had Job Last Week (Ref)	1.00		1.00	
No Job Last Week, Had Job Past 12 Mo.	1.39 (1.19, 1.62)	< .0001	1.19 (1.09, 1.30)	.0001
No Job Last Week, None Past 12 Mo.	1.08 (0.94, 1.25)	.23	1.00 (0.94, 1.07)	.84
Never Worked	0.73 (0.54, 0.99)	.05	0.55 (0.47, 0.64)	< .0001
Health Status				
Excellent (Ref)	1.00		1.00	
Very Good	1.64 (1.38, 1.95)	< .0001	1.33 (1.24, 1.43)	< .0001
Good	2.15 (1.82, 2.54)	< .0001	1.95 (1.81, 2.09)	< .0001
Fair/Poor	2.88 (2.36, 3.50)	< .0001	3.08 (2.83, 3.35)	< .0001
Psychological Distress				
No/Low Distress (Ref)	1.00		NA	
Moderate Distress	5.54 (4.91, 6.24)	< .0001	NA	NA
Serious Distress	14.50 (12.63, 16.65)	< .0001	NA	NA
Region of Residence				
South (Ref)	1.00		1.00	
NorthCentral/Midwest	1.16 (1.02, 1.32)	.02	0.90 (0.84, 0.97)	.005
Northeast	0.95 (0.82, 1.11)	.6	0.80 (0.72, 0.88)	< .0001
West	1.57 (1.38, 1.79)	< .0001	1.24 (1.15, 1.34)	< .0001
Public Coverage				
No (Ref)	1.00		1.00	
Yes	0.36 (0.31, 0.42)	< .0001	0.48 (0.44, 0.52)	< .0001
Private Coverage				
No (Ref)	1.00		1.00	
Yes	0.41 (0.36, 0.47)	< .0001	0.29 (0.27, 0.31)	< .0001

Compared to people living above 200% FPL, adults with incomes below that threshold were more likely to experience cost barriers to mental care. Specifically, individuals living below 100% FPL had 40% increased odds, and those between 100% and 199% FPL had 27% increased odds. Divorced, separated, and never married people also had higher odds of cost barriers to mental care compared to married adults. Cost barriers to dental services showed similar results to mental care. Specifically, people living below 200% poverty experienced a 97% increase in odds compared to those living above 200% FPL. Divorced and separated adults had 46% and 37% increase in odds of cost barriers to dental care post ACA compared to married adults.

The survey-weighted interaction model does not generate confidence intervals. However, Table 5 indicates that after adjusting for other variables (Table 1), post-ACA implementation was significant for adults living in the worst poverty and unmarried people indicating that these groups showed improvements post ACA. In addition, people experiencing psychological distress had significantly higher odds of experiencing cost barriers to mental services following ACA implementation. Individuals with moderate distress had OR=5.74 (p<.0001), and those with serious distress had OR=14.12 (p<.0001) compared to those with no/low distress post-ACA. Adults with self-reported health status of good and fair/poor resulted in approximately two to three times higher odds of cost barriers to health services compared to those in excellent health status. Notably, the West region of residence showed statistically significant cost barriers to healthcare services compared to the South region. Also, adults with private health insurance were more likely to experience cost barriers post ACA.

**Table 5:** Design adjusted regression model, interaction effects of major variables with pre (0) and post (1) ACA* Notes: *Adjusting for all primary variables presented in Table 1 Pre-ACA = 2011-2013; Post-ACA = 2015-2017; implementation year 2014 was excluded Abbreviations: OR = Odds Ratio, CI = Confidence Interval, Ref = Reference Category p<.05*; p<.005**; p<.0001*** Source: Author’s analysis from the National Health Interview Survey from Integrated Public Use Microdata Set (IPUMS) 2010-2017, excluding 2014

	Needed But Could Not Afford Medical Care	Needed But Could Not Afford Prescription Meds	Needed But Could Not Afford Mental Care	Needed But Could Not Afford Dental Care
	OR	OR	OR	OR
Race				
White x ACA (Ref)	1.00	1.00	1.00	1.00
Black/African American x ACA	0.95	1.02	1.05	1.02
American Indian/Alaskan Native x ACA	0.76	1.01	1.23	1.23
Asian x ACA	1.21	1.20	0.79	1.06
Other Race x ACA	1.12	0.90	0.98	1.05
Ethnicity				
Non-Hispanic x ACA (Ref)	1.00	1.00	1.00	1.00
Hispanic x ACA	1.04	1.02	1.09	1.06
Poverty Status				
>200% FPL x ACA (Ref)	1.00	1.00	1.00	1.00
100-199% FPL x ACA	1.02	1.03	0.83	0.93
<100 % FPL x ACA	0.93	0.85	0.84	0.89
Marital Status				
Married x ACA (Ref)	1.00	1.00	1.00	1.00
Widowed x ACA	1.04	0.92	1.03	1.10
Divorced x ACA	1.04	0.96	0.98	0.93
Separated x ACA	0.81	0.77	0.70	0.81
Never Married x ACA	1.01	0.94	0.96	1.01
Age				
26-34 x ACA (Ref)	1.00	1.00	1.00	1.00
35-44 x ACA	1.00	1.25*	1.00	1.04
45-54 x ACA	1.13	1.37**	0.99	1.10
55-64 x ACA	1.12	1.29**	0.89	1.27**
Gender				
Female x ACA (Ref)	1.00	1.00	1.00	1.00
Male x ACA	1.00	0.95	1.10	1.00
Educational Attainment				
No HS Diploma x ACA (Ref)	1.00	1.00	1.00	1.00
High School Grad x ACA	1.03	0.91	0.91	0.85
Some College x ACA	1.01	0.79*	0.82	0.89
College Grad x ACA	0.98	0.73*	1.00	0.81
Graduate School x ACA	1.01	0.71*	0.80	0.85
Employment				
Had Job Last Week x ACA (Ref)	1.00	1.00	1.00	1.00
No Job Last Week, Had Job Past 12 Mo. x ACA	0.86	0.95	0.65**	0.88
No Job Last Week, None Past 12 Mo. x ACA	0.86	0.96	0.73*	1.04
Never Worked x ACA	1.07	0.96	0.65	0.98
Health Status				
Excellent x ACA (Ref)	1.00	1.00	1.00	1.00
Very Good x ACA	1.00	1.01	0.78	0.87
Good x ACA	1.02	0.90	0.55**	0.89
Fair/Poor x ACA	1.17	0.97	0.33***	0.94
Psychological Distress				
No/Low Distress x ACA (Ref)	NA	NA	1.00	NA
Moderate Distress x ACA	NA	NA	5.74***	NA
Serious Distress x ACA	NA	NA	14.12***	NA
Region of Residence				
South x ACA (Ref)	1.00	1.00	1.00	1.00
NorthCentral/Midwest x ACA	0.98	1.01	1.00	0.91
Northeast x ACA	0.87	0.83	0.92	1.01
West x ACA	0.83*	0.95	1.16	0.97
Public Coverage				
No x ACA (Ref)	1.00	1.00	1.00	1.00
Yes x ACA	1.16	1.10	1.02	1.03
Private Coverage				
No x ACA (Ref)	1.00	1.00	1.00	1.00
Yes x ACA	1.34***	1.19*	1.32*	1.19*

## Discussion

The results of this study showed that people under 200% poverty, some races and Hispanic ethnicity, and unmarried adults continued to experience high odds of cost barriers to health services post-ACA although odds decreased. Poverty status and marital status showed higher odds when other variables were controlled, indicating that living under 200% poverty and being unmarried remained large barriers to care post ACA implementation from 2015 to 2017.

Poverty

Research suggests that while low and middle-income families are increasingly gaining access to health coverage, many may not receive primary healthcare services due to high deductibles and other out-of-pocket costs [15,16]. The present study supports these findings and shows that the odds of experiencing cost barriers to needed medical care for people living in poverty remained high despite the implementation of the ACA in 2014.

Studies conducted after 2014 suggest that people with mental illness who received coverage due to the ACA were still likely to encounter cost barriers to care, while poverty is linked with an increased likelihood of experiencing psychological problems and is a risk factor for exposure to trauma [17]. The results of this study support this evidence showing that people in poverty had increased odds of experiencing cost barriers to mental services post-ACA.

Poverty is a contextual factor associated with high rates of untreated dental disease and low utilization rates for U.S. adults [18]. One study found that reported financial barriers to receiving dental care were highest compared to other types of care [19]. Although reduced, the odds of cost barriers to dental services remained almost two times as high for adults under poverty compared to after ACA implementation in 2014.

The increasing cost of medications remains a challenging barrier for marginalized populations. The inability to pay for medicine is a recognized challenge with serious consequences, including preventable death [20]. Although not significant, the odds of experiencing cost barriers to prescription medications were reduced post ACA when predicting cost barrier interactions on poverty status in this study.

Race

This study data from the unadjusted logistic regression suggest that cost barrier to health services were significant for race and Hispanic ethnicity, except for Asian Americans post ACA across all services. Racial groups are disproportionately poor with consistently low rates of utilization [21]. The current study supports these findings and shows that most racial groups had approximately 40% higher odds compared to whites. However, the odds for adults of color experiencing cost barriers to health services were 14%-37% lower compared to whites in the multivariable regression model possibly indicating racial groups experienced reduced odds when adjusting for poverty status. 

After 2014, there is limited evidence suggesting significant reductions in racial disparities in mental healthcare for adults. Research shows that people of color continued to receive mental health treatment at lower rates compared to whites [22]. This does not support the results of this study of the multivariable regression model showing that except for adults of other races, individuals in all racial categories experienced a 22%-66% reduction in odds of cost barriers to services compared to whites. This could be a result of the impacts of other study variables such as income on the racial categories post ACA.

The burden of oral health disease disproportionately falls on racial minorities who have limited access to oral healthcare and low utilization rates [22]. Research suggests that racial disparities in dental care utilization among adults showed a small decrease in states with expanded Medicaid, but financial barriers continued [23]. The literature does not support the results of this study which show that the odds of experiencing cost barriers to needed dental services were 26%-45% lower for people of color (except other races). Multiple barriers to healthcare access exist for racial minorities beyond cost and can include other factors such as language, child care, geography, and cultural familiarity [24].

Race and ethnicity are also important factors in analyzing cost barriers to prescription medications. For example, African Americans historically have higher rates of difficulty in affording medications compared to white individuals (33.5% compared to 25.4%) [25]. The results of this study indicate that except for adults of other races, odds were reduced for all racial categories. Specifically, Black/African American adults had 9% reduced odds for cost barriers to medications post ACA.

Marital status

High medical bills are common in the U.S., and households without double incomes such as unmarried adults were at even greater risk for barriers and less likely to seek care [26]. The results of this study support this evidence and show that unmarried individuals had higher odds of experiencing cost barriers to medical services compared to married adults.

Especially sensitive to cost barriers, people who are unmarried often experience limited use of mental health services. Research suggests that people who are married are less likely to have unmet mental health needs [27]. The results of this study support the assertion that single people are vulnerable to cost barriers to receiving mental health services. Divorced, separated, and never married adults had higher odds of cost barriers to mental care after the ACA compared to their married counterparts.

Marital status is a known predictor of unmet oral health needs and poor service utilization for unmarried people [28]. For example, a study examining the state of Wisconsin found that rates of untreated dental needs for divorced individuals were 60%, compared to 15% for married people [29]. The results of this study support the literature and show that unmarried people had 44%-70% higher odds of experiencing cost barriers to needed dental services compared to those with a spouse post ACA.

Cost barriers to medications are challenging for unmarried people compared to those with a partner. Married people are less likely to experience cost barriers to medication and more likely to adhere to medicine requirements (63% compared to 44%) [28]. This research shows that unmarried adults had 14%-43% increased odds of experiencing cost barriers to medications compared to married adults. However, the ACA interaction analysis shows a 6%-23% decrease in odds for these marital categories after the implementation of the ACA, meaning that odds were reduced when examining the impacts of cost barriers on marital status without impacts of other variables like poverty status.

The application of Andersen’s Health Services Use model in this study suggests that the contextual and predisposing factors of the ACA implementation and coverage requirement did not significantly impact the odds of experiencing cost barriers to health services. Predisposing and enabling factors supported increased health behavior use due to ACA implementation in this instance but barriers to equitable access remained high.

It was surprising to find limited reductions and consistently high odds of experiencing cost barriers to healthcare services overall for vulnerable groups post-ACA. It was also interesting to note in the unadjusted model that Blacks/African Americans experienced between 20% and 47% higher odds of cost barriers to all services except mental care which resulted in 16% lower odds compared to whites. These results suggest that expanded eligibility and financial assistance provided by the ACA did not go far enough to statistically reduce the odds of adults under poverty, non-white adults, and unmarried individuals from experiencing cost barriers to medical, dental, mental services, and prescription medications.

Study strengths and limitations

The benefits of using the IPUMS NHIS secondary data set for health coverage analysis included no cost access to large, valid, representative data with high response rates (85%-95%). Limits to the study included recall bias issues with self-reported data; potential collection and reporting errors; and the inability to establish causal relationships. Further, other factors associated with cost barriers to services not addressed in this study could be used for outlining future research on healthcare disparities by examining other social determinates of health, such as culture, health literacy, and Medicaid expansion and non-expansion states.

## Conclusions

While structural factors are shown to contribute to the underutilization of healthcare services, those who are unmarried, living in poverty, and adults of color still reported high rates of cost barriers to needed services after the implementation of the ACA. Even though there were fewer missed healthcare services overall post ACA in 2014, living under 200% poverty remained the biggest predictor of accessing services for U.S. adults. Examining the ongoing cost barriers to achieving health equity and optimal health outcomes for individuals is necessary for assessing the impact of the ACA on marginalized groups who are most likely to face challenges.
